# The Effects of Pediatric Acute Lymphoblastic Leukemia Treatment on Cardiac Repolarization

**DOI:** 10.3390/children11101158

**Published:** 2024-09-24

**Authors:** Diana R. Lazar, Simona Cainap, Florin Leontin Lazar, Dana Maniu, Cristina Blag, Madalina Bota, Marius C. Colceriu, Mihnea Zdrenghea

**Affiliations:** 1 Department No. 11, Oncology, “Iuliu Hatieganu” University of Medicine and Pharmacy, 400012 Cluj-Napoca, Romania; maniu.diana@umfcluj.ro; 2 Department of Pediatric Cardiology, Emergency Clinical Hospital for Children, 400394 Cluj-Napoca, Romania; 3 Department of Mother and Child, “Iuliu Hatieganu” University of Medicine and Pharmacy, 400012 Cluj-Napoca, Romania; 4 Department No. 5, Internal Medicine, Medical Clinic Number 1, “Iuliu Hatieganu” University of Medicine and Pharmacy, 400012 Cluj-Napoca, Romania; 5 Biomolecular Physics Department, Faculty of Physics, “Babes-Bolyai” University, 400084 Cluj-Napoca, Romania; 6 Department of Pediatric Oncology and Hematology, Emergency Clinical Hospital for Children, 400394 Cluj-Napoca, Romania; 7 Department of Functional Biosciences, Discipline of Physiology, “Iuliu Hatieganu” University of Medicine and Pharmacy, 400012 Cluj-Napoca, Romania; 8 Department of Hematology, “Ion Chiricuta” Oncology Institute, 400015 Cluj-Napoca, Romania

**Keywords:** cardiotoxicity, childhood cancer, ECG, chemotherapy, repolarization

## Abstract

**Background**: In recent years, cardiac dysfunction in childhood cancer survivors has become an important issue. Studies are focusing on identifying means for the early identification of patients at risk. Considering this, our study aims to investigate 24-hour Holter electrocardiogram (ECG) repolarization changes throughout doxorubicin (DOX) and cyclophosphamide (CPM) administration in pediatric patients treated for acute lymphoblastic leukemia (ALL). **Methods**: This was an investigator-driven, single-center, prospective, observational study. Enrolled children had a baseline bedside ECG examination performed before starting chemotherapy (T0). Serial Holter ECG examinations were conducted at three moments during their treatment protocol: day 8 (T1), day 29 (T2), and day 36 (T3). This study evaluated several ECG repolarization parameters, such as the QT interval, corrected QT interval (QTc), and QTc dispersion, as well as ST segment variations. **Results**: We evaluated 37 children diagnosed with ALL. The T0 examination revealed that over a third of patients had a resting heart rate (HR) outside the normal range for their age and sex. During chemotherapy, statistically significant increases in both HR as well as QT and QTc dispersion values were noticed, especially during the first DOX administration. What is more, a significant increase in the percentage of patients with ST segment depression from T1 to T2 and T3 was noticed. Rhythm disturbances were rare in the study population, with only a few patients presenting ventricular or supraventricular extrasystoles. **Conclusions**: This study reveals silent repolarization changes occurring early during anticancer treatment in children treated for ALL. These findings could aid in a better understanding of the cardiac toxicity mechanism, and they could potentially improve cardiac risk stratification for oncologic patients. Because of the small number of patients, our results need to be validated by larger studies.

## 1. Introduction

Cardiac dysfunction after chemotherapy is a trending topic nowadays, with more studies dedicated to understanding its pathophysiology and the early identification of patients at risk. Cardiac toxicity following chemotherapy can lead to congestive heart failure (CHF), with a mortality rate of over 50% [1,2]. What is more, childhood cancer survivors (CCSs) often become symptomatic in their 30–40s, an age at which their lives are supposed to be most active. A CHF diagnosis at this age comes with a tremendous reduction in the quality of life, as well as important socio-economic consequences [3,4].

Acute lymphoblastic leukemia (ALL) is the most frequent pediatric malignancy, with a continuously increasing incidence. Although the 5-year survival rates have increased from 50% in the 1970s to over 90% nowadays (in high-income countries), ALL is still the most frequent cause of disease-related death in children [5]. ALL treatment consists of a polyphasic multidrug regimen including anthracyclines (ACs) and cyclophosphamide (CPM) and methotrexate (MTX) administration, which are known to cause various cardiac side effects. When compared to healthy siblings, survivors of childhood leukemia have a 4.2 times higher risk for CHF, and a 3.3 times higher risk for myocardial infarction [6].

Anthracyclines, particularly doxorubicin (DOX), have been known to cause dose-dependent cardiac toxicity, leading often to CHF [1]. It is also known that DOX has a pro-arrhythmogenic effect. Numerous cardiac electric abnormalities have been identified during and after treatment, such as a prolonged PR interval, increased QT interval, decreased QRS amplitude, and nonspecific ST segment changes [7,8]. Recent studies have shown early dysfunction in the cardiac autonomic nervous system (CANS) during DOX treatment. Particularly, studies have focused on heart rate variability (HRV) parameters as an indicator of the altered balance between the sympathetic and parasympathetic CANS. In time, this autonomic disbalance has been proven to alter cardiac contractility and increase ventricular wall stress [9,10,11].

CPM is an alkylating agent, largely known for its powerful anticancer and immunosuppressive properties. CPM cardiac toxicity is dose-dependent, with a wide spectrum of clinical manifestations from tachyarrhythmias and hypotension to CHF and fulminant myocarditis [12,13]. The incidence of CPM-induced cardiac toxicity is hard to evaluate, ranging from 8 to 20% in adult patients, and up to 5% in children. Early echocardiographic changes, such as the E/A ratio or diastolic intraventricular septum thickness, can be seen on serial evaluations [14]. However, electrocardiography (ECG) monitoring is easily available, with no inter-examiner measurement bias, and could detect early predictors of acute HF: prolonged corrected QT interval (QTc) and increased QTc dispersion (QTd) [15,16].

As mentioned, ECG is one of the most accessible, cost-efficient cardiac investigations available. Naturally, a screening process for patients at risk of developing cardiac toxicity includes an ECG evaluation. However, the electrocardiographic changes occurring during chemotherapy are heterogenous, and their role in predicting future heart function is under-evaluated. Also, a bedside ECG only evaluates a short period of cardiac electrical activity, with the patient at rest, in a supine position. A 24-h Holter ECG allows for a more complete assessment of the cardiac rhythm, by enabling an evaluation of cardiac activity during normal daily activities for both sleep and awake states, as well as during and shortly after treatment administration. On the other hand, the Holter ECG can present more movement artefacts, requiring a more thorough and lengthy interpretation period of the recorded signal. However, a study by Hegazy et al. showed that a Holter ECG evaluation in healthy children resulted in a 10% diagnostic rate of novel rhythm disturbances, underlining the efficiency of this method in screening for cardiac electrical abnormalities [17].

Current guidelines define chemotherapy-induced cardiac toxicity mostly relying on trans-thoracic echocardiographic (TTE) parameters: a novel reduction of the left ventricle ejection fraction (LVEF) to values under 40%, or a new decrease in LVEF over 10% (to values of 40–49%). Also, a smaller reduction in LVEF, accompanied by an increase in serum cardiac biomarkers, is another accepted definition [18]. Since echocardiographic changes in heart function are mostly noticeable years after chemotherapy completion, our study hypothesis was that subtle changes in heart function can be detected by an ECG evaluation during chemotherapy administration. This study aimed at evaluating ECG and Holter ECG repolarization changes during chemotherapy administration in children treated for ALL. Therefore, our study focused on evaluating cardiac repolarization parameters, such as the QT interval, QTc, QTd, and ST segment changes induced by DOX and CPM early during the oncologic treatment. Also, we evaluated heart rate (HR) and PR interval changes during this time, as well as documenting any arrhythmic episodes.

## 2. Material and Methods

The present study was an investigator-driven, single-center, prospective, observational study, aiming to document any dynamic Holter ECG changes induced by chemotherapy in children treated for ALL.

As established in our study protocol, 37 consecutives pediatric patients diagnosed with ALL were included in this study. Patients were diagnosed and treated in a tertiary pediatric hematology center in Cluj-Napoca, Romania. All children received treatment in compliance with the ALL IC-BFM 2009 protocol, including several DOX and CPM cycle administrations. Patients were enrolled from July 2019 up to July 2023, with patients still being monitored at follow-up visits. Written informed consent from every child and parent/legal guardian was obtained. Our study protocol was carried out according to the declaration of Helsinki and was endorsed by the University of Medicine and the Pharmacy “Iuliu Hatieganu” Clinical Research Ethics Committee.

Our inclusion criteria were as follows: patients had to be less than 18 years old at diagnosis, with a positive diagnosis of ALL and no history of congenital heart disease or other cardiac function abnormalities. Exclusion criteria were as follows: a LVEF below 50% before the onset of chemotherapy, prior chest radiotherapy, or a history of other potentially cardiotoxic treatments.

All patients had a baseline cardiac evaluation, prior to chemotherapy administration, consisting of a bedside ECG (T0), trans-thoracic echocardiography (TTE), high-sensitive cardiac Troponin, and NT-proBNP determination. Furthermore, all patients had serial Holter ECG measurements in the following days of the ALL IC-BFM 2009 protocol: day 8—first DOX administration (T1), day 29—fourth DOX administration (T2), and day 36—first CPM administration (T3). The T2 examination also marks the halfway point of the AC treatment, meaning a total cumulative dose of 120 mg/m^2^ for each patient. According to the ALL IC BFM 2009 protocol, following diagnosis and the first treatment protocol, patients are assigned to 3 relapse risk groups: standard-risk group (SRG), medium-risk group (MRG), and high-risk group (HRG). Patients in the HRG follow a more intensive treatment regimen later on.

The 24-hour continuous Holter ECG recordings were obtained using a 12-lead BTL HOLTER. The signal was investigated using the BTL Cardiopoint Holter H600 software. First, the recorded signal was cleared of artifacts. Furthermore, all ectopic beats and cardiac rhythm abnormalities were analyzed. The following parameters were evaluated: (i) baseline rhythm: heart rate (HR) and tachycardia and bradycardia percentages; (ii) ectopic beats: supraventricular and ventricular extrasystolic beats; (iii) atrio-ventricular conduction parameters: PR interval; and (iv) repolarization parameters: QT and QTc interval, QT and QTc dispersion, as well as ST segment depression. The absolute QT dispersion was calculated as the difference between the maximum and minimum QT and QTc values obtained on the 24-hour Holter ECG recordings. Because of very large values distribution, a relative QT and QTc dispersion was calculated, as a ratio of the calculated dispersion and the sum of the extreme QT and QTc values (QTd range = (QTmax − QTmin)/(QTmax + QTmin)) [19].

Normal ECG values were evaluated based on the nomogram published by Rijnbeek et al. Values in the 2nd–98th percentile range were considered to be normal [20]. It should be mentioned that the QTc interval was calculated using both Bazett (QT_C_ = QT/RR^1/2^) [21] and Fridericia (QT_C_ = QT/RR^1/3^) [22] formulas. The ST segment depression has been considered positive at a deflection bigger than 0.1 mV for a minimum of 30 s, measured at J point + 1/8*RR interval. Maximum ST depression values have been analyzed for each patient.

The statistical analysis was performed using Microsoft Excel 2019. The demographic data and risk group were described as number (n) and percentage (%), while the mean ± standard deviation (sd) was used to represent the data referring to patient characteristics at diagnosis (age, weight, and height), as well as ECG and Holter ECG parameters. A paired *t* test was used to assess the statistical significance of changes encountered in the evaluated ECG parameters. Also, a chi-square test was used to verify the statistical significance of changes in qualitative variables (percentages). For all employed tests, the resulting two-tailed *p*-value was interpreted to be statistically significant at a value less than 0.05.

This study’s primary endpoint was to assess changes in cardiac repolarization parameters during chemotherapy, by evaluating QT and QTc intervals, QTd, as well as ST segment variability.

## 3. Results

This study evaluated 37 children diagnosed with ALL. Most patients were between 3 and 6 years old at diagnosis, with a mean age of 5.86 ± 3.57 years old. During the study, four patients died from sepsis or progressive disease and two continued treatments abroad. There was an almost equal male to female ratio, with slightly more patients of rural origin. After the first chemotherapy cycle, most of our patients were classified as belonging to the medium-risk group (MRG), according to the ALL IC BFM 2009 protocol. All baseline patient characteristics can be seen in Table 1.

The baseline ECG examination (Table 2) revealed that more than a third of all patients had a resting HR outside the normal range for their age and sex, with the majority (83.33%) presenting HR values above the 98th percentile. Regarding the PR interval, 18.92% of patients had modified values, most of them (71.42%) with values below the second percentile for age and sex. Finally, an above normal QTc level (calculated with the Bazett formula) at diagnosis was seen in almost 19% of patients, with 8% presenting borderline values between 440 and 460 milliseconds (ms), and 11% presenting values above 460 ms. When calculating with the Fridericia method, 54.05% of QTc values were identified as outside the norm, but in this instance the majority (85%) had values below the second percentile for age and sex. No significant arrhythmia or pathological ST changes were identified.

Regarding Holter ECG parameters, changes encountered throughout treatment can be seen in Table 3.

To begin with, during the first Holter ECG (T1), 11.76% and 8.82% of patients presented with HR and PR interval values outside the normal range for their age and sex. A significant decrease in the average HR was observed when compared to the baseline ECG. The PR interval was outside the norm in up to 9% of patients, with no significant difference when compared to T0. Using the Bazett formula, QTc values above the 98th percentiles for age and sex were seen in 38.24% of patients, with the majority having borderline QTc levels. However, when applying the Fridericia formula, there were 8.82% of patients with abnormal QTc values. Nevertheless, the average QTc values (calculated with both the Bazett and Fridericia methods) presented a significant increase when compared to T0.

The second evaluation, after the fourth DOX cycle (T2), showed a significant increase in average HR values as compared to T1, and a significant increase in the minimum HR recorded. What is more, 25% of patients presented values above the 98th percentile for their age and sex. The average PR interval showed a significant increase during T2, with 12.5% of patients presenting values above the upper normal limit. Regarding the QTc interval, although the values did not differ considerably, the maximum QTc values were indeed significantly higher after the fourth DOX administration. When using the Bazett formula, 54% of patients presented values above the 98th percentile for their age and sex, with 33.3% having values above 460 ms. However, with the Fridericia method, only 12% had values above the upper normal limit, with only one patient presenting values above 460 ms.

Following the first CPM administration (T3), the average HR values continued to increase. More than half (58.33%) of the patients had HR values outside the normal range, with most of them (85.7%) presenting values above the upper normal limit. Regarding the PR interval, although the average values recorded were significantly higher than during T1, there were only 8.3% of patients with values outside the age-appropriate limits. Even though average, minimum, and maximum QTc values did not vary significantly, there were 75% (with the Bazett method) and 58.3% (with the Fridericia method) of patients with values above the 98th percentile for their age and sex.

**Figure 1:**
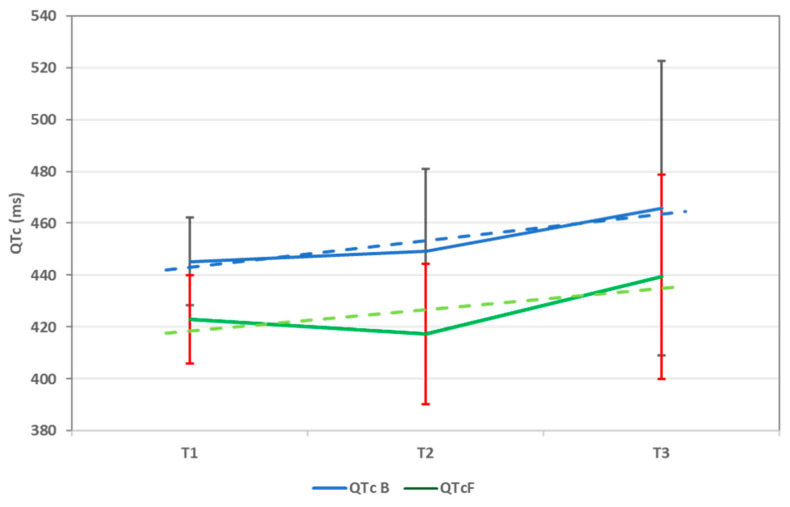
QTc trend throughout chemotherapy using both Bazett (QTc B) and Fridericia (QTc F) formulas. QTc = corrected QT interval and ms = milliseconds. Data are represented as mean ± sd.

The trend of the average QTc values (calculated using both formulas), from T1 to T3, can be seen in Figure 1.

Also, it should be mentioned that the tachycardia percentage continued to increase significantly from T1 to T2 and T3, respectively. On the other hand, while the bradycardia percentage considerably decreased from T1 to T2, it displayed an increase during T3, although it was not statistically significant (*p*-value: 0.564).

QT and QTc interval dispersion values (Table 4) increased from T1 to T2. QTc dispersion changes (based on the QTc interval corrected using the Bazett formula) from T1 to T2 were found to be statistically significant. The relative QT dispersion (QT dispersion range) showed a significant increase from T1 to T2, followed by a decrease during T3.

**Figure 2:**
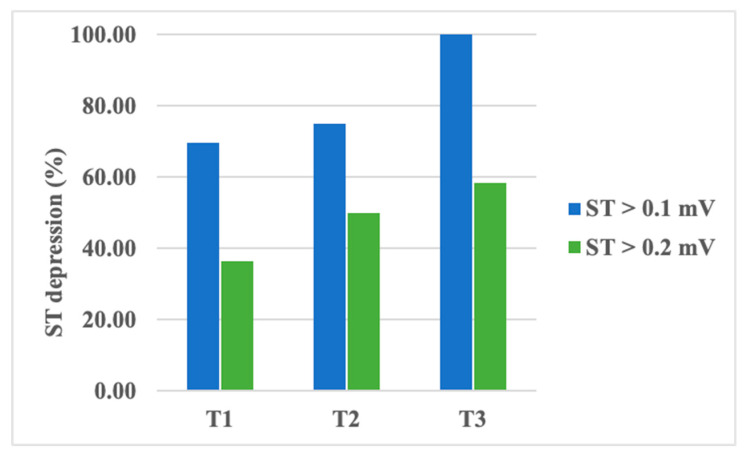
Percentage of patients presenting transient ST segment depression during chemotherapy.

An analysis of ST segment depression values showed a decrease in amplitude from T1 to T3 without statistical significance (Table 5). However, a transient depression of the ST segment, higher than 0.1 mV, was recorded in up to 70% of patients during T1, almost 75% of patients during T2, and in all of our patients during T3. When evaluating ST segment depressions higher than 0.2 mV, the same pattern was noticed: up to 37% of patients during T1, 50% during T2, and 58.3% during T3 (Figure 2). The observed increase in transient ST segment depression frequency was found to be statistically significant, with a *p*-value of 0.04 (using the chi-square test). However, ST segment variability did not vary significantly between the examinations.

**Figure 3:**
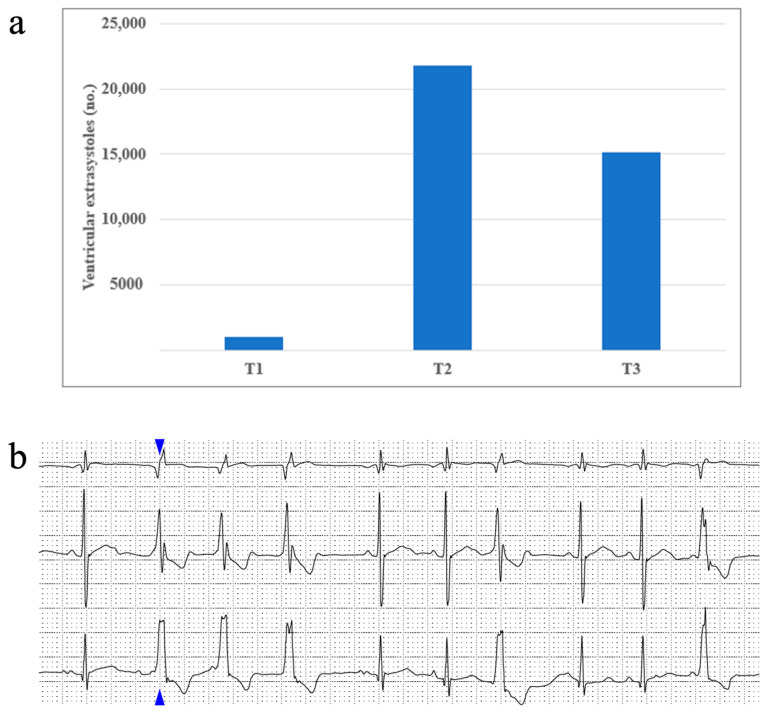
(**a**) One patient’s ventricular extrasystoles evolution during treatment and (**b**) an image taken from the same patient’s Holter ECG recording, displaying an example of ventricular extrasystoles organized as triplets (the blue arrow shows the first extrasystole of this set, followed by another two identical ones).

Rhythm disturbances occurred scarcely in our study population. About half of our patients presented sinus arrhythmia, and up to 8% presented a wandering atrial pacemaker. Supraventricular extrasystoles occurred in 32% of patients during T1, 45% of patients during T2, and only 8% of patients during T3. Ventricular extrasystoles were identified in up to 13% of patients at T1, 25% of patients at T2, and almost 16% of patients in T3. No episodes of sustained or non-sustained ventricular tachycardia were identified in our study. We report one patient who had a maximum of 21809 (16%) monomorphic ventricular extrasystoles with a right bundle block appearance and tendency of organization: 3004 couplets, 31 triplets, 2640 bigeminy beats, and 3680 trigeminy beats being identified during the studied period. Figure 3 portrays this patient’s ventricular extrasystole evolution from T1 to T3.

## 4. Discussions

In a continuous search of the early markers of cardiotoxicity, subtle ECG changes during chemotherapy have gained increased attention. A bedside ECG is a simple examination, performed quickly, with little to no measurement bias (compared to trans-thoracic echocardiography). It can easily identify arrhythmias occurring during chemotherapy, ischemic changes, or any other more subtle variations that can precede arrhythmia onset. Still, the information provided by a bedside ECG is limited to the surveyed moment and it could miss transient electrical abnormalities or arrhythmia episodes. More interestingly, Holter ECG changes can be tracked throughout chemotherapy and can give insight into subtle daily changes in heart function. However, data on Holter ECG changes during chemotherapy, particularly in children, are still limited.

Our study used a 24-hour Holter ECG to evaluate changes in heart electrophysiology in pediatric patients treated for ALL. The main findings of this study are as follows: (1) DOX treatment induces an increase in HR values and tachycardia percentage, from the very first cycle of chemotherapy; (2) the PR interval lengthens upon DOX and CPM treatment; (3) QT, QTc values, and QTc dispersion, increase with DOX treatment, highlighting its proarrhythmogenic potential; and (4) ST segment depression amplifies with ongoing chemotherapy. This study was limited by the small number of patients enrolled, with further larger studies being needed to validate our findings.

### 4.1. HR Changes on Chemotherapy

Recent years have seen an increase in studies evaluating heart rate variability (HRV) during chemotherapy, particularly during AC treatment. It has been shown that a decrease in HRV parameters develops from the first doses of DOX, and it persists throughout treatment. HRV illustrates the function of the CANS, and, therefore, a decrease in its parameters points out a sympathetic hyperactivity, which could be one of the first signs of AC-induced cardiac toxicity [11,23].

While it has not been the purpose of this manuscript to investigate HRV parameters, an increased sympathetic activity has also been noticed in our study. Although a significant decrease in HR values was noticed during T1, compared to baseline, this can be credited to the 8-day cortisone treatment prior to chemotherapy initiation. Steroid therapy is known to induce sinus bradycardia as a side effect [24]. This has been the case in our study, as an important bradycardia percentage was detected during T1. Following T1 examination, as the DOX cumulative dose increased and prednisone dose decreased, the HR values and tachycardia percentage significantly increased, while the bradycardia percentage decreased, respectively, illustrating the predominant sympathetic activation of the CANS during AC treatment. In a systematic review of the literature, Bertrand et al. concluded that HR abnormalities occur commonly in childhood ALL survivors [25]. Our study emphasizes that these alterations in heart function begin very early, from the first days of chemotherapy.

### 4.2. PR Interval on Chemotherapy

The PR interval is representative of atrio-ventricular conduction, with a prolonged PR interval indicative of a atrio-ventricular blockage of various degrees. During chemotherapy, particularly AC treatment, the PR interval has been shown to increase with a higher cumulative dose [25,26,27]. Our study also confirms this theory, as the PR interval values continued to increase significantly, from the first to the fourth DOX dose, as well as during and after the first CPM administration.

There are few studies to date evaluating conduction disorders in CCSs, which indeed show an increased prevalence of major atrio-ventricular conduction disturbances [26,27]. Still, none of our patients developed atrio-ventricular conduction blocks during the exanimated period. If the PR interval trend observed in our study persists, it is likely that seriate evaluations, particularly after chemotherapy completion, will identify, in some patients, the development of atrio-ventricular conduction blocks.

### 4.3. Repolarization Abnormalities

Studies evaluating QT interval changes induced by chemotherapy have shown significant changes occurring in the immediate hours to days following AC treatment. QT interval prolongation, a known marker of repolarization disorders [28,29], is not to be overlooked, as it can progress to polymorphic ventricular tachycardia (torsade de pointes) and sudden cardiac death. The QT interval is influenced by the HR, hence a correction of the QT interval according to the HR is needed. Most often used, the Bazett formula tends to over-diagnose long QTc at an increased HR. This is why, in situations like these or for evaluating children with long QT syndrome (LQTS), the Fridericia formula is considered to be more accurate [30,31]. The Holter ECG evaluation has been shown to be an increasingly sensible method of detection [32,33], with recent studies involving a Holter QTc analysis in the risk stratification of HF patients [34]. The Friedericia formula is utilized in the majority of research that employ the Holter ECG to evaluate the QTc interval in children to identify LQTS [35]. Studies on the QTc interval in CCS patients did not find a statistically significant increase following the end of chemotherapy; nonetheless, there is evidence linking QTc prolongation to LV dysfunction in these patients [36,37].

In our study, we decided to use both formulas for calculating QTc to evaluate the impact of both methods on long QTc detection. To begin with, the bedside evaluation (T0) was very representative for how the Bazett formula can lead to the overdiagnosis of long QT. After calculating with the Fridericia method, the QTc levels were lower, and fewer surpassed 460 ms. Next, we evaluated QTc using a Holter ECG at given time points during chemotherapy (T1, T2, and T3). We noticed that, compared to T0, average QTc values (using both formulas) were higher during T1, with a high statistical significance. What is more, the average values continued to increase during following examinations, although not significantly. This confirms that chemotherapy, particularly DOX, leads to an early increase in the QTc interval. Also, as expected, the Bazett formula has been proven to increase long QTc detection in our study, with values frequently above 460 ms, which did not verify when calculating with the Fridericia method. What is more, during the third examination (T3), we detected many patients with increased QTc values above 460 ms (using both formulas), underlining the effect CPM has on the QTc length.

Lately, QT dispersion (the difference between the maximum and minimum QT intervals in a 12-lead electrocardiogram) has been studied as a novel marker for arrhythmia risk and sudden death [38,39]. According to the Rotterdam study, it is being viewed as an important predictor of cardiac mortality in adults and elderly patients [40]. Increased QTd indicates enhanced heterogeneity within the heart, an aspect that could favor arrhythmogenesis [41]. Furthermore, studies have shown an increase in QTc dispersion during and after AC and CPM treatments [16,42]. In their study, Nakamae et al. found that QTc dispersion is an independent risk factor strongly associated with acute HF following premedication for hematopoietic stem cell transplantation [15]. In children, QTc dispersion has also been used as a predictor for mortality, as Shabestari et al. revealed in their study on ill preterm newborns [43]. In another study, Turan et al. showed an increased QTc dispersion in children with multisystem inflammatory syndrome (MIS-C), signaling the increased risk for acute ventricular arrhythmia in this setting [44]. In adults, normal values vary widely, ranging from 10 to 80 ms in healthy subjects, and studies have shown little to no age or sex-related differences [45].

In our study, the QTd values obtained were high (even higher for QTc dispersion) compared to normal values encountered in the literature. However, until now, QTc dispersion has rarely been evaluated via a Holter ECG [33], particularly in children receiving chemotherapy; therefore, we believe that the normal values described on a bedside ECG cannot be applied here. To correct the extreme variability of the QT values detected, we calculated a relative QT dispersion—QT dispersion range. When compared, the relative QTd presented a highly significant increase from T1 to T2 (following DOX administration), and then a slight significant decrease during T3 (on CPM administration). This highlights the impact DOX has on cardiac repolarization, favorizing a repolarization heterogeneity and therefore an increased arrhythmia risk.

### 4.4. ST-T Changes on Chemotherapy

Nowadays, computer data analysis techniques have become increasingly sophisticated, yielding Holter ECG recordings to be analyzed in depth. However, regarding the ST segment evaluation, measuring methods and the interpretation on the Holter ECG still lacks consensus. Many studies have chosen various methods of defining ST segment variations—from choosing a different definition of the baseline level to utilizing a different measurement moment, referred to as the J point [46,47]. Also, one of the main challenges with interpreting ST segment changes on a Holter ECG is identifying and eliminating positional changes. This aspect is even more challenging in children, who have an increased movement rate, even during sleep.

In patients with coronary syndromes, episodes of asymptomatic ischemia are frequently encountered, with some studies reporting a prevalence of up to 50%. The monitoring of such episodes by a Holter ECG has provided us with a better understanding of their pathophysiology and their significance [47]. Most studies have shown that the presence of asymptomatic ischemia during ECG monitoring corelates with an adverse cardiac prognosis. Boon et al. evaluated 194 hypertensive adult patients with an ambulatory ECG Holter to detect transient ST segment changes. The first software-based evaluation of ST segment variation led to a more than double prevalence of silent ischemia in their cohort. Therefore, they concluded that an automated ST analysis, however objective, can overestimate ST segment variation; consequently, producing more strict analysis criteria or a secondary manual checkpoint would be needed to improve the detection accuracy [48].

In children, ST segment depression on a Holter ECG has been scarcely investigated. However, Harahsheh et al. reported a case of silent ischemia revealed by a Holter ECG, in a child with complex heart surgery and a normal bedside ECG [49]. Also, Hamed et al. reported increased ST segment inversions episodes detected by a Holter ECG in children with β-thalassemia major [50].

In our study, although ST depression amplitudes did not vary significantly in between examinations, a significant increase in the number of patients presenting positive ST depressions has been identified. This is of extreme importance, as it shows an ischemic preconditioning of the cardiac tissue early during chemotherapy treatment. Further evaluations of patients would be required to uncover the relationship between ST depression during chemotherapy administration and further HF development.

Regarding arrhythmia incidence, our study did not find an increased occurrence of major arrhythmic events during treatment. The majority of our patients presented sinus arrhythmia and, a small percentage of them, a wandering atrial pacemaker, which is normally encountered with variations in children [51,52]. However, an increase in sinus tachycardia was noticed, particularly during doxorubicin administration. This confirms the link between doxorubicin administration and an increase in sympathetic activation [10]. Also, our study reported an increase in ventricular extrasystole frequency from T1 to T2, followed by a decrease at T3. Considering that from T1 to T2 patients continue to receive DOX (four doses), this finding highlights the powerful arrhythmogenic properties DOX has during a short-term period. CPM administration, during T3, did not seem to exhibit the same potent effect.

## 5. Limitations

The present study was limited to a small cohort of patients from a single center, with an even smaller number expected at the end of the study, partly due to a difficult adherence of patients to the study protocol. A larger, multi-centric study is needed to confirm our findings. Furthermore, several Holter ECG examinations are needed to continue our research, carried out at the end of chemotherapy, after 1- and 2-years following treatment completion, to better assess the ECG parameters trend through the entire treatment, as well as any lingering end-treatment changes. What is more, none of the patients presented any early signs of chemotherapy-induced cardiotoxicity and, as it usually takes more than a decade for patients to become symptomatic or present echocardiographic signs of cardiotoxicity, our study should be interpreted more like a hypothesis-generating study, as the clinical significance of our Holter ECG findings is still to be observed in the long-term follow-up.

## 6. Conclusions

This study reports that silent repolarization changes occur as early during the anticancer treatment as the first DOX dose, presenting an aggravating tendency with following cycles of AC. To begin with, our results have shown a significant increase in repolarization heterogeneity following DOX administration, which in turn results in an increased arrhythmia risk. Also, ST segment changes during the early cycles of chemotherapy uncover a silent ischemic preconditioning of the myocardium. This could represent a starting point for cardiac function changes detected in childhood cancer survivors. These results emphasize the need for continued research on the impact that these subclinical ECG changes have on heart function in the long run. These results could help improve cardiac risk stratification for the oncological patient, and, therefore, more appropriate secondary prevention measures. However, a long-term patient follow-up is needed to better understand the clinical significance of these findings.

## Figures and Tables

**Table 1:** Patient characteristics at diagnosis.

Characteristics	Mean ± sd	n (%)
**Age (years)**	5.86 ± 3.57	
1–3 years		7 (18.92%)
3–6 years		16 (43.42%)
6–10 years		9 (24.32%)
10–18 years		5 (13.51%)
**Gender**		
male		19 (51.35%)
female		18 (48.65%)
**Origin**		
urban		17 (45.95%)
rural		20 (54.05%)
**Weight (kg)**	23.71 ± 13.28	
**Height (m)**	1.17 ± 0.24	
**Relapse risk group**		
Standard-risk group		2 (5.41%)
Medium-risk group		23 (62.16%)
High-risk group		12 (32.43%)

**Table 2:** Baseline bedside ECG parameters.

ECG Parameters (T0)	
HR (bpm)	101.76 ± 27.24
PR interval (ms)	118.08 ± 19.26
QTc interval (ms) Bazett	423.41 ± 36.85
QTc interval (ms) Fridericia	385.37 ± 30.96

HR = heart rate; QTc = corrected QT interval; and ms = milliseconds. Data are shown as mean ± sd.

**Table 3:** Holter ECG parameters during chemotherapy.

	T1	T2	T3	*p*-Value ^a^	*p*-Value ^b^	*p*-Value ^c^
**HR (bpm)**						
minimum	53.88 ± 11.87	65.5 ± 20.77	70.5 ± 21.07	**-**	**0.016**	**0.001**
average	86.24 ± 13.57	106.5 ± 19.01	107.25 ± 27.3	**0.001**	**<0.001**	**0.001**
maximum	160.71 ± 25.27	169.83 ± 21.27	168.58 ± 28.09	-	0.141	0.261
**Tachycardia (%)**	6.4 ± 10.75	20.29 ± 27.59	34.74 ± 29.97	**-**	**0.022**	**0.005**
**Bradycardia (%)**	29.91 ± 25.41	7.75 ± 15.71	14.5 ± 26.52	**-**	**<0.001**	**0.010**
**PR interval (ms)**						
minimum	87.82 ± 12.21	92.08 ± 10.9	101.33 ± 17.35	-	0.374	**0.017**
average	122.41 ± 18.57	136.67 ± 20.88	135 ± 19.76	0.084	**0.008**	**0.049**
maximum	206.65 ± 57.6	218.33 ± 58.2	207 ± 55.5	-	0.281	0.822
**QT interval (ms)**						
minimum	292.85 ± 41.92	268.15 ± 46.24	292.75 ± 41	-	0.235	0.582
average	377.35 ± 30.36	347.75 ± 38.58	369.25 ± 53.75	**<0.001**	**<0.001**	0.229
maximum	447.65 ± 43.07	446.5 ± 50.12	465.83 ± 68.47	-	0.772	0.847
**QTc interval (ms) Bazett**						
minimum	376.06 ± 35.78	372.54 ± 45.07	375.5 ± 75.02	-	0.699	0.923
average	445.21 ± 16.89	449.21 ± 31.72	465.75 ± 56.78	**<0.001**	0.484	0.244
maximum	568 ± 36.41	597.08 ± 43.21	593.25 ± 45.65	**-**	**0.024**	0.128
**QTc interval (ms) Fridericia**						
minimum	281.31 ± 43.48	272.35 ± 47.62	303.50 ± 43.64	-	0.777	**0.045**
average	422.96 ± 17.08	417.31 ± 26.94	439.42 ± 39.46	**<0.001**	0.369	0.324
maximum	618.82 ± 55.65	628.58 ± 59.32	650.96 ± 72.71	-	0.552	0.552

HR = heart rate; bpm = beats per minute; ms = milliseconds; and QTc = corrected QT interval. Data are shown as mean ± SD. ^a^ Comparison between T0 and T1; ^b^ comparison between T1 and T2; and ^c^ comparison between T1 and T3. All *p*-values were assessed using a paired *t* test; *p*-values < 0.05 were considered statistically significant; and significant *p*-values were written in bold.

**Table 4:** QTc dispersion during chemotherapy.

Parameters	T1	T2	T3	*p*-Value ^i^	*p*-Value ^ii^
**QT d (ms)**	151.94 ± 74.83	178.35 ± 57	173.08 ± 71.66	0.5653	0.6646
**QTc d (B) (ms)**	191.94 ± 49.53	224.54 ± 45.04	217.75 ± 92.59	**0.001**	0.542
**QTc d (F) (ms)**	337.51 ± 87.44	356.22 ± 82.31	347.46 ± 95.04	0.886	0.283
**relative QTd**	19.15 ± 4.93	25.26 ± 9.48	22.65 ± 8.27	**0.025**	0.432

QTd = QT dispersion; QTc = corrected QT interval using the Bazett (B) or Friedricia (F) formula; ms = milliseconds. Data are shown as mean ± sd. ^i^ Comparison between T1 and T2 and ^ii^ comparison between T1 and T3. All *p*-values were assessed using a paired *t* test; *p*-values < 0.05 were considered statistically significant; and significant *p*-values were written in bold.

**Table 5:** Maximum ST segment depression during chemotherapy.

	T1	T2	T3
**ST depression**	0.35 ± 0.58	0.36 ± 0.42	0.29 ± 0.20
**ST segment variability**	1.01 ± 1.07	1.01 ± 0.91	0.84 ± 0.44

Data are shown as mean ± sd.

## Data Availability

The data presented in this study are available on request from the corresponding author. The data are not publicly available because of GDPR legislation.
